# Influenza and pertussis vaccination during pregnancy – attitudes, practices and barriers in gynaecological practices in Germany

**DOI:** 10.1186/s12913-019-4437-y

**Published:** 2019-09-02

**Authors:** Stefanie Böhm, Marianne Röbl-Mathieu, Burkhard Scheele, Michael Wojcinski, Ole Wichmann, Wiebke Hellenbrand

**Affiliations:** 1 Immunization Unit, Robert Koch Institute, Berlin, Germany; 2 Standing Committee on Vaccination, Robert Koch Institute, Berlin, Germany, Munich, Germany; 3 German Professional Association of Gynaecologists, Munich, Germany; 4 Working Group Immunization, German Professional Association of Gynaecologists, Munich, Germany

## Abstract

**Background:**

In Germany, antenatal influenza vaccination is recommended since 2010, but uptake remains low. Several countries recently introduced antenatal pertussis vaccination, which is currently under consideration in Germany. We conducted a survey among gynaecologists on attitudes, practices and barriers regarding influenza and pertussis vaccination during pregnancy.

**Methods:**

Gynaecologists were invited to complete a pre-tested, 24-item questionnaire published in the German Professional Association of Gynaecologists’ journal in September 2017 within 2 months. Associations between variables were examined using Chi-Squared, Fischer’s Exact or t-tests. Variables associated with gynaecologists’ self-reported implementation of vaccination in pregnant women were identified using univariate and multivariate logistic regression analyses.

**Results:**

Of 867 participants (response 11%), 91.4 and 59.4% reported currently vaccinating pregnant women against influenza and pertussis, respectively. Gynaecologists who reported obtaining annual influenza vaccination and actively informing their patients about these vaccinations were significantly more likely to vaccinate pregnant women against influenza (96.5% vs. 65.7 and 95.1% vs. 62.2%) and pertussis (63.1% vs. 44.3 and 82.4% vs. 12.9%). Performing influenza vaccination was least likely among gynaecologists who perceived logistical difficulties as a vaccination barrier (35.9%), while pertussis vaccination was least likely if the lacking official recommendation (32.0%), logistical difficulties (27.1%), safety concerns (17.5%) and limited vaccine effectiveness (11.1%) were perceived as barriers. Of participants not yet vaccinating pregnant women against pertussis, 86.5% reported they would follow an official recommendation. Including vaccination recommendations in the maternity record (95.2%) and informing the public (88.7%) and health care professionals (86.6%) were considered the most suitable measures to achieve high pertussis vaccination coverage.

**Conclusions:**

The large proportion reporting performance of influenza vaccination during pregnancy and high acceptance of a potential recommendation for pertussis vaccination reflected positive attitudes towards vaccination among participants. However, factors associated with failure to vaccinate may be more prevalent among non-participants. Results suggest that gynaecologists’ confidence in vaccination is crucial for implementing vaccination in pregnancy. Thus, doubts on vaccine effectiveness and safety should be allayed among gynaecologists and pregnant women via various communication channels, and solutions for logistical barriers sought. Including antenatal vaccination recommendations in the maternity record would serve as an important reminder for both groups.

**Electronic supplementary material:**

The online version of this article (10.1186/s12913-019-4437-y) contains supplementary material, which is available to authorized users.

## Background

### Influenza vaccination during pregnancy

In Germany, 5 to 20% of the population contracts seasonal influenza annually [1]. In several studies pregnant women had more severe disease than other adults [2–4], with a 7- to 9-fold increased risk for hospitalisation during the 2009 influenza pandemic [5, 6] as well as later influenza seasons [7]. Infants are also at increased risk for severe disease and complications such as otitis media and pneumonia [2]. Therefore, in 2010, the German Standing Committee on Vaccination (STIKO) recommended influenza vaccination for pregnant women from the second trimester of pregnancy, or in case of an underlying chronic disease, from the first trimester onwards [2, 8], with the goal of protecting mothers as well as infants. Influenza vaccines are not licensed for infants until 6 months of age. Thus, maternal vaccination aims to reduce the risk of transmission to the infant from the mother (cocooning) on the one hand and confer passive immunity in the first weeks of life on the other. A recent review of randomized clinical trials found evidence that maternal vaccination reduced the incidence of laboratory confirmed influenza both in mothers and their infants [9]. Despite these benefits, according to the most recent data available from Germany, influenza vaccination uptake remained low at 11 to 23% in women who were pregnant during the influenza seasons of 2012/13 and 2013/14 [10–13]. This was similar to the 24% median vaccination coverage for the influenza season 2014/15 among eight reporting EU member states, but lower to the reported coverage in the United Kingdom (44–56%) [14].

### Pertussis in infants and pertussis vaccination during pregnancy

As in many western countries [15], the pertussis disease burden in Germany remains substantial despite high vaccination coverage in children [16, 17]. This is at least partly explained by lower effectiveness of currently available acellular vaccines versus previously available whole cell vaccines [18] and rapidly waning immunity [19]. Incidence is highest in infants too young to be vaccinated, who are also at highest risk for life-threatening complications, such as pneumonia, seizures, pulmonary hypertension and hypoxic encephalopathy [15, 20, 21]. A recent study in Germany during a period of lower disease activity in 2013–2015 estimated the incidence of pertussis requiring hospitalization in infancy at 50 cases/100,000 infants [22]. In epidemic years, this number is likely to be 2 to 3 times higher. From 2013 to 2016, 58% of all infants with pertussis reported to the national communicable disease notification system and 83% of those younger than 3 months of age were reported to be hospitalized [17]. Of 7 pertussis-related deaths reported from 2013 to 2017, 3 were in infants (case fatality: 0.2%), the remainder in persons over 60 years of age [23].

Pertussis vaccination is recommended by STIKO for all infants from 2 months of age [24]. Unless vaccinated in the last 10 years, STIKO recommends pertussis vaccination for women of child-bearing age, women postpartum and close household contacts of infants (preferably by 4 weeks before birth) to protect young infants from pertussis (cocoon strategy) [25]. However, only 23% of pregnant women in a large nationwide cross-sectional survey in 2013 [11] and 22% of household contacts of infants interviewed as part of a large population based telephone survey in 2012/13 reported having obtained a pertussis vaccination within the last 10 years, the latter an increase of 11% from 2009/2010 [26, 27]. In addition, several studies have shown limited impact of cocooning strategies on infant pertussis disease burden [28–32].

Pertussis vaccination of pregnant women has recently been introduced in several countries. Antenatal maternal vaccination is associated with efficient transfer of pertussis-specific antibodies from mother to infant [33, 34] and several studies have shown high effectiveness ranging from 69 to 95% for preventing pertussis in the first 2–3 months of life [35–43]. Several systematic reviews [33, 34, 44–46] concluded that administration of tetanus, diphtheria, pertussis (Tdap) vaccine in pregnancy is safe, although the quality of the evidence in underlying studies is mainly low. Since late 2016, the summary of product characteristics for two pertussis-containing vaccines available in Germany were updated with data on the safety administration in pregnancy; similar updates are planned for other products [17]. Tdap vaccination of pregnant women is not yet recommended in Germany, but STIKO is currently evaluating the evidence for a potential recommendation according to its standard procedure [47]. In Germany, gynaecologists in private practice (i.e. all practicing physicians in the field of Gynaecology and Obstetrics in outpatient care, as opposed to gynaecologists working in hospital care) are the primary health care providers for pregnant women and thus instrumental in providing maternal vaccinations, which is not the task of midwives. In view of currently low influenza vaccination coverage in pregnant women and a possible STIKO recommendation for pertussis vaccination of pregnant women, the Robert Koch Institute (RKI) conducted a survey of gynaecologists in private practice in collaboration with the German Professional Association of Gynaecologists (BVF) and their working group on vaccination. Existing literature on health care workers’ attitudes about influenza vaccination in pregnancy may differ between countries due to heterogeneous vaccination programs and health systems, and little is known regarding attitudes about pertussis vaccination during pregnancy. Assessing attitudes and acceptability specifically in gynaecologists, with their role as the main implementer, is important in view of a potential pertussis vaccination recommendation. Our goal was to assess gynaecologists’ attitudes and practices with respect to influenza and pertussis vaccination and to identify perceived barriers and opportunities for their implementation.

## Methods

### Study population and questionnaire

Our target population included all gynaecologists in private practice. In Germany, every practicing physician must be affiliated with one of the State Chambers of Physicians [48]. According to the statistics of the German Medical Association (BÄK), containing the combined data of the State Chambers of Physicians, there were 11,500 gynaecologists in private practice in 2016 in Germany [49].

We developed a 24-item questionnaire (Additional file 2) by focussing on the study aim and taking previous experience and literature findings into account [50]. After an internal pre-test by colleagues of the Immunization Unit at the RKI for comprehensibility and time required (approximately 8 min), the questionnaire was sent to elected officials of the BVF and their working group on vaccination (in total approx. 150 persons) in July 2017. In total, we received feedback from 43 persons. The questionnaire covered awareness and implementation of current vaccination recommendations. We also asked about possible vaccination barriers and what measures gynaecologists might consider effective for achieving high pertussis vaccination coverage in pregnant women in case of a STIKO recommendation. Additional items covered general vaccination practices and demographics. Questions were mainly closed-ended with yes/no answer options, and we specified the use of other categories in the results. We grouped agreement with vaccination barriers into “agree” (rather or fully agree), “partly agree” and “disagree” (rather or fully disagree) and suitability of measures into “suitable” (very suitable and rather suitable), “partly suitable”, “not suitable” (rather not suitable or not suitable at all) and “unsure”. Free text comments were permitted to state additional barriers for influenza or pertussis vaccination during pregnancy as well as to suggest further measures to achieve high pertussis vaccination coverage in pregnant women.

### Data collection and sample size calculation

As a way of gaining access to a large proportion of the target population, the questionnaire was published along with background information in the 2017 September edition of the monthly BVF journal *Frauenarzt* (*“Gynaecologist”*) that is circulated to all its members, including approximately 8000 gynaecologists in private practice. Thus, we could potentially reach about 70% of our target population through the BVF access. The authors are not aware of differences between gynaecologists in private practice with and without BVF membership. Through the collaboration with the BVF and with the goal of achieving high participation, we were able to additionally distribute the questionnaire through the *BVF@ktuell* newsletter (5164 subscribers), the online platform Gyn-Netz (1850 subscribers) and the BVF’s federal state representatives, all of which also addressed only the potentially reachable target population of BVF members. In order to avoid multiple participation of persons who may have been invited several times to take part in the survey, we have expressly pointed out that only one participation per person is desired. No incentives were offered for participation in the survey. A reminder was published in the October edition of *Frauenarzt*. Participants could complete the survey on paper or using the online platform Voxco (Voxco Version 5.5.1.205). Paper questionnaires were to be sent to the RKI anonymously via mail, scanned via e-mail, or by fax. Data collection was closed on November 6th.

At a confidence level of 95%, the minimum number of participants needed from the target population of 11,500 gynaecologists to determine a proportion of 50% at an absolute precision of 5 and 3%, was calculated to be 372 and 977, respectively, using OpenEpi. https://www.openepi.com/SampleSize/SSPropor.htm

### Statistical analysis

Responses of paper questionnaires were double-entered. In our descriptive analyses we displayed categorical variables as percentages. To evaluate representativeness we compared demographic characteristics of our participants to BÄK statistics on all privately practicing gynaecologists. We used the Chi Square Test or Fischer’s Exact Test to test for associations between categorical variables and the t-test to compare continuous variables within categories of a second variable, for influenza and pertussis vaccination, respectively. We performed stratified analyses to further assess potential associations of performing vaccination with demographics, other vaccination practices and perceived barriers. Moreover, univariate and multivariate logistic regression analyses (adjusted for age, sex and region) were conducted to identify variables associated with the implementation of influenza and pertussis vaccination in pregnant women, by comparing gynaecologists who currently do vaccinate to those who do not, respectively for the two vaccinations. *P*-values < 0.05 were considered statistically significant. We categorized text responses to open questions by creating keywords and assigning each response. We performed statistical analysis using Stata® version 14 (StataCorp, Texas, USA) and used Microsoft Excel (2010) to create figures.

## Results

### Response and description of participants

We received 934 questionnaires, 734 (79%) through the online platform and 200 (21%) paper-based.

**Fig. 1:**
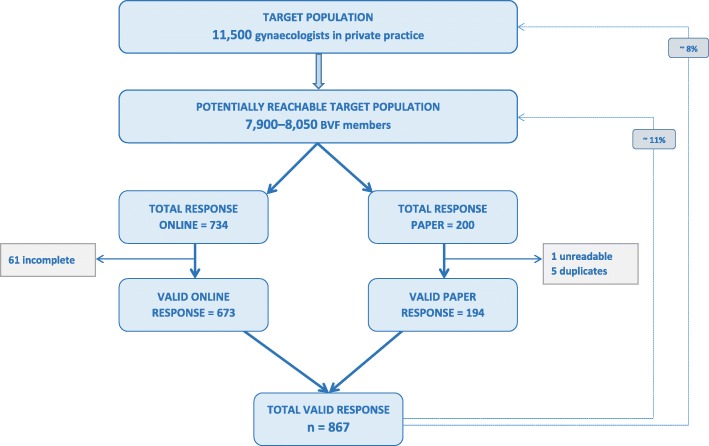
Response of privately practicing gynaecologists in Germany for participation in our survey

**Fig. 2:**
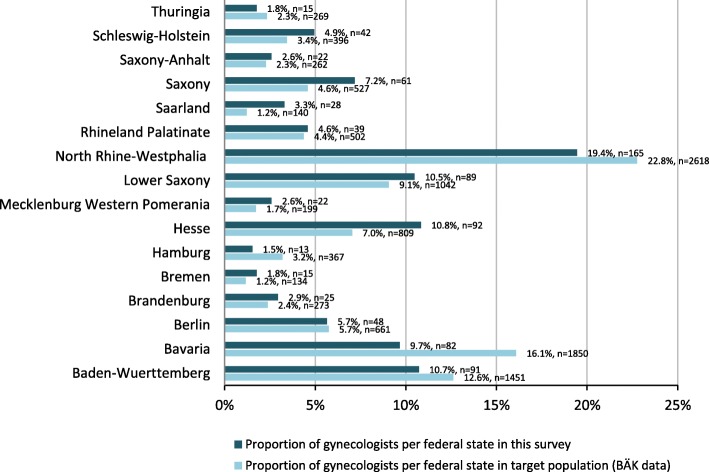
Distribution of survey participants versus all privately practicing gynaecologists registered with BÄK across federal states

Since there was no individual login function for the online survey, we excluded incomplete questionnaires to avoid double counting of persons who logged in several times without completion. Unreadable or duplicate questionnaires sent by scan or fax were also not taken into account. As shown in Fig. 1, we were able to include 867 questionnaires in the final analysis. Thus, approximately 11% of BVF members participated, corresponding to 8% of all gynaecologists in private practice (Fig. 1). Compared to all privately practicing gynaecologists registered with the BÄK, a higher proportion of survey participants was female, aged 50–59 years and from eastern federal states; a lower proportion was 60 years or older and from western federal states (Table 1). At the federal state level, gynaecologists from Bavaria, North Rhine-Westphalia and Hamburg were underrepresented in our survey, while those from Saarland, Hesse and Saxony were overrepresented (Fig. 2). Participants had spent a median of 15 years in private practice (IQR: 9–22). When asked about their own influenza vaccination practices, 70.6% reported annual vaccination, 16.6% occasional vaccination and 12.8% never obtaining vaccination. The proportion of gynaecologists reporting regular influenza vaccination was higher in eastern than western federal states (82.1% vs. 68.1%, *p* = 0.004), but did not differ according to age, sex or years of work experience.

**Table 1:** Characteristics of survey participants compared to all privately practicing gynaecologists registered with BÄK [49]

		Survey (*n* = 867)		BÄK (*n* = 11,500)
Characteristics		Frequency	Percent	Percent
Sex		856		
Female		653	76.3	66.3
Male		203	23.7	33.7
Age (in years)		842		
≤49		277	32.9	31.8
50–59		419	49.8	41.1
≥60		146	17.3	27.1
Work experience in gynaecological practice (in years)		854		
≤9		217	25.4	–
9–19		346	40.5	–
≥20		291	34.1	–
Geographical region		849		
East^a^		145	17.1	13.3
West^b^		704	82.9	86.7

Abbreviations: *BÄK* German Medical Association (Bundesärztekammer)

^a^East: Brandenburg, Mecklenburg Western Pomerania, Saxony, Saxony-Anhalt, Thuringia

^b^West: Baden-Wuerttemberg, Bavaria, Berlin, Bremen, Hamburg, Hesse, Lower Saxony, North Rhine-Westphalia, Rhineland Palatinate, Saarland, Schleswig-Holstein

Missing values were not considered, thus the number of participants differs slightly between variables

### Influenza vaccination in gynaecological practices

#### Current practice of influenza vaccination during pregnancy

**Fig. 3:**
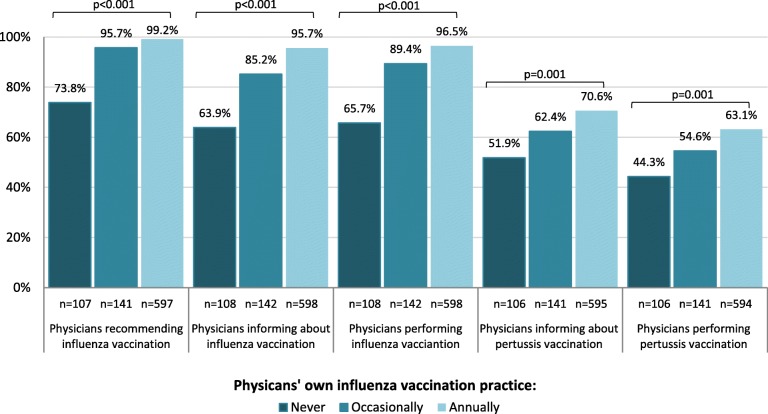
Proportion of gynaecologists who stated recommending, actively informing their pregnant patients or performing influenza and pertussis vaccination, according to their own influenza vaccination practices

Almost all respondents were aware of the influenza vaccination recommendation for pregnant women (99.2%) and most stated recommending (95.4%) and performing (91.4%) it. Although the majority reported informing their pregnant patients about this recommendation (98.5%), 8.6% of them informed on patient request only. Compared to gynaecologists who actively informed patients, the latter were significantly less likely to state recommending (65.3% vs. 99.1%, *p* < 0.001) and performing vaccination (62.2% vs. 95.1%, p < 0.001) or to provide information material on influenza vaccination during pregnancy in their practice (18.9% vs. 65.0%, p < 0.001). Among gynaecologists who offered their pregnant patients influenza vaccination, 44.2% stated that ≥50% women accepted vaccination (Fig. 6). This proportion was higher among gynaecologists who actively informed pregnant women (47.0% vs. 3.9%, p < 0.001). While we did not find a significant association between recommending or performing influenza vaccination and sex, age, years of work experience, or geographical region, there was a strong association with participating gynaecologists’ own influenza vaccination practices (Fig. 3; Additional file 1: Table S1).

#### Perceived barriers for influenza vaccination during pregnancy

**Fig. 4:**
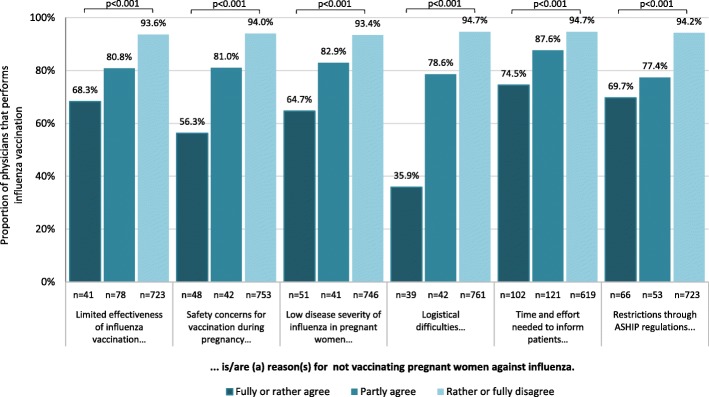
Proportion of gynaecologists performing influenza vaccination in pregnant women, in relation to their agreement with possible barriers for implementation

Participants were asked if six possible barriers for influenza vaccination of pregnant women applied to them, as outlined in Fig. 4 and listed in Additional file 1: Table S1. They had the opportunity to state additional barriers. Only a small proportion acknowledged the listed items to be vaccination barriers, but those who did were significantly less likely to report vaccinating their pregnant patients (Fig. 4). This was confirmed in both univariate and multivariate logistic regression analysis (Additional file 1: Table S1). Overall, time and effort required to inform pregnant women was most often perceived as a barrier (26.4% at least partly agreed). However, physicians who perceived difficulties in integrating vaccination into routine practice processes as a barrier were least likely to report actually performing vaccination (Fig. 4). The exclusive availability of large package sizes for influenza vaccines was mentioned as one such factor in additional comments (Additional file 1: Table S2). Billing regulations are defined at the federal state level by the Association of Statutory Health Insurance Practitioners (ASHIP). Of our participants, only 7.8% agreed that billing restrictions were an obstacle for influenza vaccination during pregnancy, but this proportion was higher among gynaecologists affiliated with the ASHIP Bavaria (13.6%), ASHIP Saxony (19.0%) and ASHIP Rhineland Palatinate (26.3%). Among participants who perceived ASHIP restrictions to be a vaccination barrier, the proportion reporting to recommend influenza vaccination to pregnant patients was lower (87.7% vs. 96.1% among all others, *p* = 0.002), and the proportion reporting to perform vaccination lower still (69.7% vs. 93.0%, among all others *p* < 0.001; Additional file 1: Table S1). Physicians who did not inform or only informed pregnant patients of the vaccination recommendation upon request (*n* = 87) more often agreed that the time and effort required for consultation constituted a barrier for vaccination (44.2% vs. 8.5% among all others, *p* < 0.001). In additional comments (Additional file 1: Table S2), some gynaecologists indicated that pregnant women often held misconceptions about vaccination during pregnancy which frequently led to vaccination refusal, despite time-consuming consultation. They also claimed that remuneration was insufficient for the time and effort expended. Gynaecologists who stated to not recommend influenza vaccination (*n* = 38) more frequently agreed that limited effectiveness (50.0% vs. 2.6% among all others, p < 0.001) and safety concerns (57.9% vs. 3.2% among all others, p < 0.001) were barriers. To a lesser extent, this also held for participants who reported never obtaining influenza vaccination themselves, among whom a higher proportion agreed that limited effectiveness (21.2% vs. 2.6% among all others, p < 0.001), safety concerns (22.1% vs. 3.4% among all others, p < 0.001) and low perceived risk for severe disease in pregnant women (20.2% vs. 4.1% among all others, *p* < 0.001) were barriers for influenza vaccination of pregnant women.

### Pertussis vaccination in gynaecological practices

#### Current practice of present pertussis vaccination recommendations

**Fig. 5:**
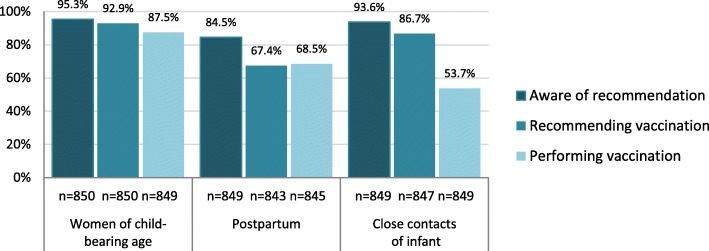
Proportion of gynaecologists who stated being aware of, recommending and performing pertussis vaccination for the following groups, if no vaccination was obtained in the previous 10 years: women of child-bearing age, women postpartum and close infant contacts

Most gynaecologists were aware of the current pertussis vaccination recommendations targeting women of child-bearing age and close infant contacts (Fig. 5). Although the majority (86.7%) stated recommending pertussis vaccination to close infant contacts, only 53.7% stated performing this in their practice. Over one third (39.4%) agreed that billing restrictions for vaccination of infant contacts through ASHIP regulations were a barrier. This proportion was higher in western than eastern federal states (42.0% vs. 27.7%, *p* = 0.001) and highest among gynaecologists affiliated with ASHIP Baden-Württemberg (55.1%), Bavaria (72.0%) and Berlin (72.3%). Although these physicians stated recommending vaccination of close contacts as often as others, they were less likely to report performing this in their practices (26.1% vs. 72.5% among all others, *p* < 0.001).

#### Current practice of pertussis vaccination during pregnancy

**Fig. 6:**
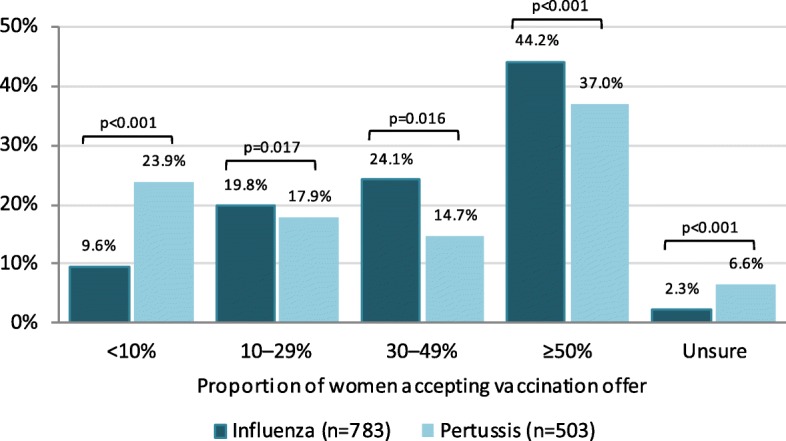
Distribution of gynaecologists’ estimation for the acceptance of influenza and pertussis vaccination of pregnant women, respectively. For example, 9.6% of gynaecologists in our survey reported that less than 10% of their pregnant patients accepted an influenza vaccination following a vaccination offer

Of participating gynaecologists, 698 (82.1%) stated informing their patients about the possibility of pertussis vaccination during pregnancy. Of these, 18.6% informed upon patient request only. Physicians who obtained annual influenza vaccination were more likely to inform pregnant women about pertussis vaccination (Fig. 3). Over half of participants (59.4%) reported already vaccinating pregnant women against pertussis despite a lacking STIKO recommendation. This proportion was non-significantly higher in eastern federal states (64.3% vs. 58.2%, *p* = 0.18), and highest among participants from Saxony at 76.3%. Physicians who actively informed patients were more likely to report vaccinating than those who did not or only upon patient request (82.4% vs. 12.9%, *p* < 0.001; Additional file 1: Table S3). Pertussis vaccination of pregnant patients was not significantly associated with age, work experience or region overall (Additional file 1: Table S3). Male gynaecologists stated more frequently to perform pertussis vaccination during pregnancy compared to female gynaecologists, which was statistically significant in western (67.1% vs. 55.4%, *p* = 0.007), but not in eastern federal states (68.0% vs. 63.6%, *p* = 0.67). Physicians who stated implementing cocoon strategy recommendations (Additional file 1: Table S3) and those who obtained influenza vaccination themselves were more likely to report performing pertussis vaccination in pregnancy (Fig. 3; Additional file 1: Table S3). About a quarter (23.9%) of participants reported that only < 10% and over a third (37.0%) that ≥50% of pregnant patients accepted their pertussis vaccination offer (Fig. 6). Among physicians who did not actively inform patients, fewer reported an acceptance of ≥50% (5.6% vs. 39.4%, *p* < 0.001).

#### Perceived barriers for pertussis vaccination during pregnancy

**Fig. 7:**
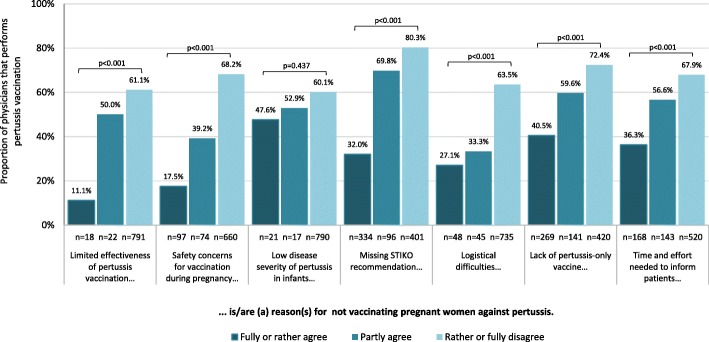
Proportion of gynaecologists performing pertussis vaccination in pregnant women, in relation to their agreement with possible barriers for implementation

As for influenza vaccination, participants were asked if seven possible barriers for pertussis vaccination of pregnant women applied to them, as shown in Fig. 7 and Additional file 1: Table S3. They were also asked if additional barriers applied. Overall, a higher proportion of gynaecologists agreed that barriers existed for pertussis compared to influenza vaccination during pregnancy, and those who did were less likely to report informing about or performing pertussis vaccination. This association was confirmed in both univariate and multivariate logistic regression analysis for all proposed barriers except for low perceived disease severity in infants (Additional file 1: Table S3). Participants most often agreed that the lack of a STIKO recommendation was a barrier for pertussis vaccination of pregnant patients (40.1%: 67.8% among physicians who reported not yet vaccinating pregnant women vs. 21.6% among all others (p < 0.001)), followed by lack of a pertussis-only vaccine (32.2%) and the time and effort needed to inform patients (19.9%). Physicians who agreed that the lack of a STIKO recommendation, limited vaccine effectiveness, safety concerns and logistical difficulties were vaccination barriers were least likely to state performing pertussis vaccination (Fig. 7; Additional file 1: Table S3). As for influenza vaccination, physicians commented that fear or scepticism of vaccination during pregnancy often led to refusal, despite thorough explanation of benefits (Additional file 1: Table S4). Physicians also commented that the lack of a pertussis-only vaccine required additional explanation and some thought recent vaccination with a tetanus-containing vaccine was problematic. Other comments addressed limited availability of pertussis-containing vaccines, concerns regarding possible long-term effects on the unborn child and fear of potential legal consequences (Additional file 1: Table S4).

The majority of participants not yet vaccinating against pertussis during pregnancy stated they would routinely recommend and perform pertussis vaccination if STIKO were to recommend this (86.5%). However, this proportion was significantly lower among those who agreed that limited effectiveness (56.3%), safety concerns (72.8%) and a low risk posed by infant pertussis (27.3%) were vaccination barriers.

#### Measures for attaining high pertussis vaccination coverage in pregnant women

**Fig. 8:**
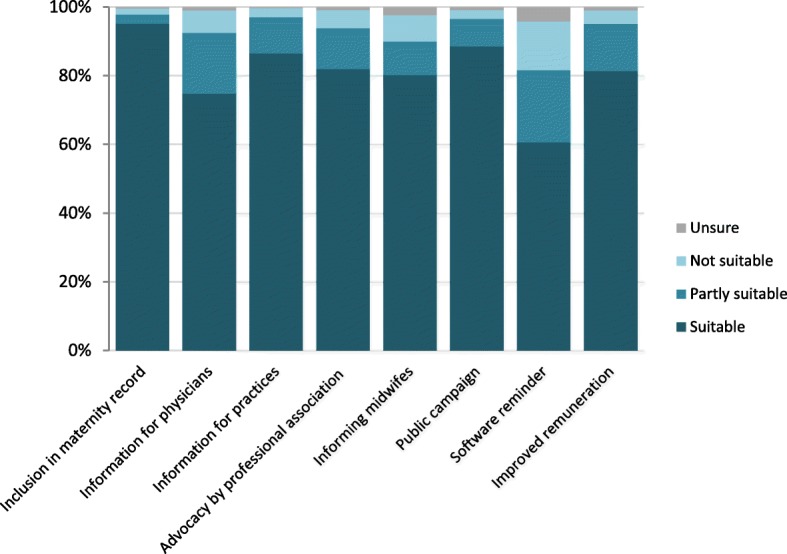
Gynaecologists’ ratings of the suitability of suggested measures to attain high pertussis vaccination coverage in pregnant women (*n* = 849–856)

Participants were asked to rate the suitability of 8 possible measures for attaining a high pertussis vaccination coverage in pregnant women in the case of a STIKO recommendation (Fig. 8). They could state additional measures. Almost all participants (95.2%) considered the integration of the recommendation into the maternity record issued to all women for the documentation of health care during pregnancy a suitable measure to attain high pertussis vaccination coverage. The majority also rated information campaigns and material such as flyers or posters for practices suitable (88.7 and 86.6%, respectively). This also held for educating midwives on vaccinations, improved remuneration for informing about and performing vaccinations, as well as an advocating position by the professional association for gynaecologists (80.3 to 82.0%). Providing information material for physicians and vaccination reminders through the practice software were rated suitable less frequently (74.8 and 60.7%, respectively). In additional comments, participants proposed better information and continuing education of health care professionals, including general practitioners, midwives and practice support staff (Additional file 1: Table S5). Many comments addressed how best to inform pregnant patients. Using a range of media to convey positive messages, including non-scientific media such as TV, campaigns, the internet, social media/apps and educational settings, was suggested. Others proposed removing various prior stated barriers, the use of incentives and vaccination reminders. Some comments related to improving vaccination coverage in general or that of close contacts, e.g. through mandatory vaccination.

## Discussion

This is the first survey in Germany to assess attitudes towards and performance of pertussis vaccination to protect mothers and their infants among privately practicing gynaecologists. Although a survey on attitudes towards and recommendation of influenza vaccination for pregnant women was performed previously [50], this did not assess active informing and actual performance of vaccination.

### Attitudes towards influenza and pertussis vaccination among gynaecologists

The majority of gynaecologists participating in our survey was aware and supportive of current recommendations for influenza and pertussis vaccination for pregnant women or, in case of pertussis, their contacts or women of child-bearing age. Strong support for influenza vaccination in pregnancy was also reported in a previous German survey [50]. However, this is not reflected in high vaccination uptake by pregnant women in Germany [10–13]. In another recent German survey, pregnant women rated gynaecologists’ attitudes towards vaccination in pregnancy as only moderate; only 54% of surveyed women were aware of the recommendation themselves and 44% stated this as the reason for not having obtained the vaccine [13]. Possible explanations for these discrepancies include poor communication between physicians and patients or socially desirable responses in the surveys. For instance, in a recent US study all participating gynaecologists stated recommending influenza vaccination, yet only 85% of their pregnant patients stated having received a recommendation [51]. However, our low response and biased participation likely also play an important role.

Gynaecologists in our survey reported higher acceptance for influenza compared to pertussis vaccination among pregnant women (Fig. 6). In countries that have implemented pertussis vaccination in pregnancy, in contrast, acceptance was reported to be higher for pertussis vaccination [52, 53]. We suspect this difference could be due to the lack of an official recommendation and subsequent lower awareness for pertussis vaccination in pregnancy at the time of our survey. However, our and other findings show that the cocoon strategy currently in place to prevent pertussis infections in infants also lacks implementation [11]. Bödeker et al. showed that pregnant women in Germany rated the risk of pertussis infection for children low [13]. This highlights the importance that awareness about pertussis vaccination recommendations need to be increased in both gynaecologists and the targeted population of recommendations.

### Determinants and barriers for offering vaccination

The Complacency, Convenience and Confidence (“3Cs”) model by the SAGE working group describes vaccine hesitancy as a complex interplay of many different factors [54]. While the model was developed primarily to explain vaccine hesitancy among target groups for vaccination, the 3Cs can also be applied to vaccinators and we highlight this in the subsequent discussion.

Physicians who obtained influenza vaccination themselves were far more likely to actively inform about and vaccinate their pregnant patients against both influenza and pertussis in our survey, as also observed by others [50, 52, 55, 56]. In our survey, concerns of limited effectiveness and safety were more common among gynaecologists who reported not obtaining annual influenza vaccination. Thus, physicians’ own vaccination practices seem to reflect their confidence in vaccination [55]. Additionally, those who obtained annual influenza vaccination were more likely to provide information material on vaccination in their practice. Not surprisingly, for pertussis, the lack of a STIKO recommendation was perceived as a barrier for performing pertussis vaccination by a high proportion of participants. Indeed, the majority of participants said they would offer this vaccination to pregnant patients if recommended by the STIKO. This suggests a fundamental acceptance of Tdap vaccination and trust in STIKO decisions. Despite these encouraging findings among our participants, vaccine hesitancy was identified among a minority (likely to be larger among non-participating gynaecologists) who perceived low vaccine effectiveness, vaccine safety concerns or (the misconception) that influenza or pertussis pose only a low risk for pregnant women and infants to be vaccination barriers. However, the latter – a complacency-related barrier – was significantly associated only with performing influenza but not pertussis vaccination (Additional file 1: Table S1 and S3). These findings underline the importance of a transparent decision process on the part of the STIKO and the need to inform health care professionals about the evidence for vaccination recommendations, also to address doubts or misbeliefs that lead to hesitancy.

Convenience-related barriers included the time and effort needed to inform pregnant women about vaccination, which were frequently perceived as a vaccination barrier, especially among gynaecologists who reported they informed only upon patient request. In additional comments, participants related this to scepticism regarding vaccination on the part of pregnant women. Similarly, a recent German survey found that pregnant women commonly believed influenza vaccination was more harmful than influenza infection [13]. Some participants claimed that women refused vaccination despite extensive consultation. This could lead to frustration and decrease motivation to recommend and perform vaccination. A few participants commented that negative media coverage led to low vaccination acceptance, and this is corroborated by a representative survey that found pregnant women refused vaccination due to vaccine-critical reports or advice from family and friends [10]. Another study found that a high proportion of elderly participants (35%) reported their decision to obtain influenza vaccination was based on the recommendation of family or friends [57].

Only a small proportion of gynaecologists perceived the integration of influenza and pertussis vaccination into their practice procedures to be barriers. However, this was associated with a much lower likelihood of performing the respective vaccinations in pregnant women and is likely to be more common among non-participating gynaecologists. Thus, convenience measures such as ensuring availability of smaller package sizes and prevention of vaccine shortages could facilitate implementation of vaccination. Remuneration is another convenience-related factor, and gynaecologists who reported billing restrictions through ASHIP regulations for influenza vaccination in pregnant women or, more commonly, pertussis vaccination in close infant contacts were less likely to state performing vaccinations. This may contribute to the poor implementation of the cocoon strategy. While billing restrictions had a large impact on performing vaccination, they had minimal (influenza) or no impact (pertussis) on recommending vaccination. However, uptake of influenza vaccination in pregnant women is much lower if only recommended, but not performed [58].

Over a third of our participants perceived the lack of a pertussis-only vaccine to be a barrier to some extent, with a resultant lower likelihood of performing the vaccination. Availability of such a vaccine as recently licensed in Thailand [59] and studied in Switzerland [60] would therefore likely additionally increase acceptance and coverage [61].

### Possible measures to achieve high vaccination uptake in pregnant women

Our findings suggest several approaches to improve implementation of vaccination recommendations by gynaecologists and to support them in their important role as vaccination advocates.

Almost all participants thought the inclusion of vaccination recommendations in the maternity record held by pregnant women would help attain high pertussis vaccination coverage. The maternity record acts as an inventory for the documentation of recommended tests and procedures in the ante-, peri- and postnatal period. In a recent survey on acceptance of vaccination in England, pregnant women proposed such a checklist to ensure reception of all recommended aspects of pregnancy care, including vaccination [62]. The inclusion of vaccination recommendations is already under discussion with the Federal Joint Committee (G-BA) responsible for issuing the maternity record.

In Germany, remuneration for vaccination is currently linked to the administration of a vaccination. Some participants in our survey felt compensation for vaccination services was insufficient, particularly the lack of compensation for vaccination consultation not linked to administration of vaccines. In keeping with this, the majority of participants agreed that improved remuneration would be an appropriate measure to achieve high pertussis vaccination coverage.

On the other hand, only 61% of participants in our survey thought recall systems were a useful measure, although they have shown to be efficient at increasing vaccination information, acceptance and coverage [63–65]. A possible explanation may be that pregnancy is not a regularly recurrent event and thus, implementation of software reminders may be difficult. Further reasons for this are unclear and should be investigated.

Our findings strongly suggest that, in view of gynaecologists, public knowledge and awareness need to be increased for both influenza and – in case of a STIKO recommendation – pertussis vaccination in pregnancy. A large majority of participants rated public campaigns and information material for pregnant patients in practices as suitable measures for achieving high pertussis vaccination coverage. Using social media or apps was also suggested. Such approaches may reduce concerns and hesitancy in pregnant women and could help facilitate consultation on vaccination by physicians, as also suggested by results of a survey among paediatricians [66].

Physicians who took the initiative to actively inform pregnant women about vaccination were less likely to doubt vaccine effectiveness or have safety concerns and more likely to obtain vaccination themselves or to report high uptake among their pregnant patients. A recent EU report on vaccine confidence points out that confidence in vaccination is crucial to achieve high vaccination coverage [67]. Thus, increasing knowledge and therefore confidence in these vaccinations among gynaecologists appears crucial in achieving high influenza and, in case of a recommendation, pertussis vaccination uptake among pregnant women. Three quarters of participants thought that information material specifically for physicians, including general practitioners, could help attain high vaccination coverage. While this seems plausible – particularly for a new vaccination recommendation targeting a vulnerable group such as pregnant patients – we are unaware of evidence showing that provider education improves vaccination uptake. Such material should address the benefits and limited risks of recommended vaccinations, as well as harms due to vaccine preventable diseases. This could be provided by the STIKO, for instance through its vaccination app, as well as through professional associations such as the BVF.

Over half the participants thought education of midwives could also encourage vaccination uptake. This is supported by other studies: In a German survey, pregnant women rated midwives’ attitudes towards vaccination in pregnancy as only moderate [13]. Others showed that, compared to gynaecologists, midwives were less aware of vaccination recommendations [68] and had more safety concerns about vaccination during pregnancy [69]. Engaging midwives and providing information based on their needs was found to be effective [70], and involving them in an immunization programme led to higher vaccination coverage in pregnant women in Australia [71]. Although midwives are not involved in delivering vaccination in Germany, they play a significant role in antenatal and postnatal care and thus, their support of vaccination recommendations is important for a successful implementation.

### Limitations

Our survey had several limitations, the most important being the low response of only 11%. This was despite our invitation to participate through various channels to maximize awareness for the survey and to address as many members of the target population as possible, accepting that some might be contacted more than once, and despite offering multiple response options. A recent survey on influenza and HPV vaccination achieved a slightly higher response of 20% by directly sending questionnaires to privately practicing gynaecologists [50]. Although about 70% of privately practicing gynaecologists receive the BVF journal *Frauenarzt*, the proportion of active readers may be lower. Nonetheless, demographic characteristics did not differ markedly between survey participants and all privately practicing gynaecologists. Although female gynaecologists were slightly over- and the oldest group underrepresented, sex and age did not have a large impact on our findings. Participation was higher among gynaecologists from eastern federal states, where vaccination acceptance and coverage has consistently been higher for most vaccines, both in physicians and the general population [27, 50, 72–74]. This also held for Saxony, where pertussis vaccination in pregnancy is already recommended by a state-based immunization technical advisory group. To account for these differences, rooted in very different development of vaccination regulations in the two parts of Germany prior to re-unification in 1989 [50, 75], we compared these regions in our analyses. Influenza vaccination coverage among gynaecologists in our survey (70.6%) was higher than reported from a telephone survey of gynaecologists in private practice (50.4%) [73]. However, in a more recent survey that used self-administered questionnaires, reported coverage was similar at 72.2% [50]. Nonetheless, it seems likely that highly motivated gynaecologists committed to vaccination were over-represented in our survey. This likely explains the high proportion of participants reporting pertussis vaccination of their pregnant patients despite the lacking STIKO recommendation. Therefore, non-participating gynaecologists may be more likely to question effectiveness and safety of vaccines in pregnancy or to consider the efforts required to implement vaccination – related to information of patients, logistics or to remuneration – as not worthwhile.

As discussed above, our findings identify important barriers for the implementation of influenza and pertussis vaccination during pregnancy. Thus, findings provide information on how implementation of current vaccination recommendations by gynaecologists could be improved and high pertussis vaccination uptake among pregnant women could be achieved, should STIKO recommend this in the future.

## Conclusions

Despite the limitations of our survey, we gained valuable insight into current vaccination practices of gynaecologists in private practice in Germany. The majority of our participants stated their willingness to offer pertussis vaccination to their pregnant patients should this be recommended by the STIKO. Our results suggest the implementation of vaccination recommendations targeting diseases in mothers and their infants is associated with physicians’ own vaccination uptake and an active approach to vaccinations, which are both likely linked to their confidence in vaccination. Therefore, we recommend enhancing the focus and inclusion of vaccination in continuing medical education activities to counter potential uncertainties with available scientific evidence for vaccine effectiveness and safety and to emphasize and support physicians’ role as vaccination advocates. The integration of antenatal vaccination recommendations into the maternal record as part of official standard procedures during pregnancy care would serve as an important reminder for both physicians and pregnant women. Means to remove barriers such as billing restrictions and logistical challenges should also be sought. Gynaecologists themselves stressed the importance of increasing patient awareness and dispelling vaccine scepticism through the use of a wide range of media including information material in practices.

## Additional files

Additional file 1: **Table S1.** Results of logistic regression analysis of variables potentially associated with vaccinating pregnant women against influenza. **Table S2.** Additional perceived barriers for vaccinating against influenza during pregnancy provided in participants’ additional comments. **Table S3.** Results of logistic regression analysis of variables potentially associated with vaccinating pregnant women against pertussis. **Table S4.** Additional perceived barriers for vaccinating against pertussis during pregnancy provided in participants’ additional comments. **Table S5.** Measures to attain high pertussis vaccination coverage in pregnant women (and high vaccination coverage in general) provided in participants’ additional comments. (DOCX 37 kb)

Additional file 2: Translated questionnaire (originally used in German) used for this survey. (PDF 138 kb)

## Data Availability

Since participants were informed that results would be published in aggregate form only, we are unable to publicly share the raw dataset. However, the data are available from the corresponding author on reasonable request.

## Abbreviations

ASHIP: Associations of Statutory Health Insurance Physicians (Kassenärztliche Vereinigung, KV)
BÄK: German Medical Association (Bundesärztekammer)
BVF: German Professional Association of Gynaecologists (Berufsverband der Frauenärzte e.V.)
CI: Confidence interval
G-BA: Federal Joint Committee (Gemeinsamer Bundesausschuss)
IfSG: German Infection Protection Act (Infektionsschutzgesetz)
IQR: Inter-quartile range
OR: Odds ratio
RKI: Robert Koch Institute
STIKO: Standing Committee on Vaccination (Ständige Impfkommission)
Tdap: Tetanus, diphtheria, pertussis
