# Supplementary material for: Influenza and pertussis vaccination during pregnancy – attitudes, practices and barriers in gynaecological practices in Germany
Source: BMC Health Serv Res. 2019 Sep 2;19:616. doi: 10.1186/s12913-019-4437-y (PMC6719372; doi:10.1186/s12913-019-4437-y)
Supplement: Supplementary file 1 — Table S1. Results of logistic regression analysis of variables potentially associated with vaccinating pregnant women against influenza. Table S2. Additional perceived barriers for vaccinating against influenza during pregnancy provided in participants’ additional comments. Table S3. Results of logistic regression analysis of variables potentially associated with vaccinating pregnant women against pertussis. Table S4. Additional perceived barriers for vaccinating against pertussis during pregnancy provided in participants’ additional comments. Table S5. Measures to attain high pertussis vaccination coverage in pregnant women (and high vaccination coverage in general) provided in participants’ additional comments. (DOCX 37 kb) [file 12913_2019_4437_MOESM1_ESM.docx]

Table S1: Results of logistic regression analysis of variables potentially associated with vaccinating pregnant women against influenza

|  |  | | N (%) | Crude OR  [95%-CI] | | p-value | Adjusted^a^ OR  [95%-CI] | p-value |
| --- | --- | --- | --- | --- | --- | --- | --- | --- |
| Being aware of STIKO influenza recommendation for pregnant women | | 858 | | |  |  |  |  |
|  | No | | 7 (0.8%) | Ref | |  |  |  |
|  | Yes | | 851 (99.2%) | 4.33 [0.82─22.70] | | 0.083 |  |  |
| Informing pregnant women about influenza vaccination | | | 859 |  | |  |  |  |
|  | No | | 13 (1.5%) | Ref | |  |  |  |
|  | Only on patient request | | 74 (8.6%) | 2.63 [0.78─8.83] | | 0.118 |  |  |
|  | Always | | 772 (89.9%) | **30.91** [9.65─98.98] | | 0.000 |  |  |
| Recommending influenza vaccination to pregnant women | | | 856 |  | |  |  |  |
|  | No | | 39 (4.6%) | Ref | |  |  |  |
|  | Yes | | 817 (95.4%) | **22.53** [11.18─45.39] | | 0.000 |  |  |
| **(A) reason(s) for not vaccinating pregnant women against influenza during pregnancy is/are …** | | | | | | |  |  |
| …doubts on the effectiveness of the influenza vaccination | | | 842 |  | |  |  |  |
|  | Fully or rather agree | | 41 (4.9%) | **0.15** [0.07─0.30] | | 0.000 | **0.16** [0.08─0.34] | 0.000 |
|  | Partly agree | | 78 (9.3%) | **0.29** [0.15─0.54] | | 0.000 | **0.31** [0.16─0.60] | 0.000 |
|  | Rather or fully disagree | | 723 (85.9%) | Ref | |  | Ref |  |
| ...concerns regarding the safety of the influenza vaccination during pregnancy | | | 843 |  | |  |  |  |
|  | Fully or rather agree | | 48 (5.7%) | **0.08** [0.04─0.16] | | 0.000 | **0.08** [0.04─0.16] | 0.000 |
|  | Partly agree | | 42 (5.0%) | **0.27** [0.12─0.62] | | 0.002 | **0.35** [0.14─0.88] | 0.026 |
|  | Rather or fully disagree | | 753 (89.3%) | Ref | |  | Ref |  |
| …the low health risk posed by influenza in pregnant women | | | 838 |  | |  |  |  |
|  | Fully or rather agree | | 51 (6.1%) | **0.13** [0.07─0.25] | | 0.000 | **0.14** [0.07─0.27] | 0.000 |
|  | Partly agree | | 41 (4.9%) | **0.34** [0.14─0.81] | | 0.015 | **0.38** [0.15─0.97] | 0.042 |
|  | Rather or fully disagree | | 746 (89.0%) | Ref | |  | Ref |  |
| ...difficulties integrating the influenza vaccination into practice procedures | | | 842 |  | |  |  |  |
|  | Fully or rather agree | | 39 (4.6%) | **0.03** [0.02─0.06] | | 0.000 | **0.03** [0.01─0.06] | 0.000 |
|  | Partly agree | | 42 (5.0%) | **0.20** [0.09─0.45] | | 0.000 | **0.19** [0.08─0.43] | 0.000 |
|  | Rather or fully disagree | | 761 (90.4%) | Ref | |  | Ref |  |
| …the amount of time necessary to inform about the influenza vaccination | | | 842 |  | |  |  |  |
|  | Fully or rather agree | | 102 (12.1%) | **0.16** [0.09─0.29] | | 0.000 | **0.16** [0.09─0.28] | 0.000 |
|  | Partly agree | | 121 (14.4%) | **0.40** [0.21─0.76] | | 0.005 | **0.38** [0.20─0.73] | 0.004 |
|  | Rather or fully disagree | | 619 (73.5%) | Ref | |  | Ref |  |
| ...restrictions due to ASHIP regulations | | | 842 |  | |  |  |  |
|  | Fully or rather agree | | 66 (7.8%) | **0.14** [0.08─0.26] | | 0.000 | **0.12** [0.06─0.23] | 0.000 |
|  | Partly agree | | 53 (6.3%) | **0.21** [0.10─0.43] | | 0.000 | **0.18** [0.09─0.38] | 0.000 |
|  | Rather or fully disagree | | 723 (85.9%) | Ref | |  | Ref |  |
| Sex | | | 849 |  | |  |  |  |
|  | Female | | 646 (76.1%) | Ref | |  |  |  |
|  | Male | | 203 (23.9%) | 0.88 [0.51─1.53] | | 0.658 |  |  |
| Age (in years) | | | 835 | 0.99 [0.96─1.02] | | 0.634 |  |  |
| Work experience in gynaecological practice (in years) | | | 847 | 1.00 [0.98─1.04] | | 0.628 |  |  |
| Geographical region | | | 842 |  | |  |  |  |
|  | West | | 698 (82.9%) | Ref | |  |  |  |
|  | East | | 144 (17.1%) | 2.04 [0.92─4.55] | | 0.080 |  |  |
| Influenza vaccination practice of physician | | | 848 |  | |  |  |  |
|  | Never | | 108 (12.7%) | Ref | |  |  |  |
|  | Occasionally | | 142 (16.8%) | **4.41** [2.26─8.59] | | 0.000 |  |  |
|  | Annually | | 598 (70.5%) | **14.32** [7.94─25.82] | | 0.000 |  |  |

Abbreviations: CI = confidence interval, ASHIP = Associations of Statutory Health Insurance Physicians, OR = odds ratio, STIKO = Standing Committee on Vaccination; n = 827 in adjusted analysis

^a^ adjusted for age, sex, region (east/west)

Table S2: Additional perceived barriers for vaccinating against influenza during pregnancy provided in participants’ additional comments

| **Categorized barriers** | N |
| --- | --- |
| **Total** | **126** |
| Medical reasons:  Contraindications (mostly acute infections and allergies)  Former complications  (Non-recommended) stage of pregnancy | 42  26  7  10 |
| Refusal of pregnant women | 33 |
| Hesitancy of physicians based on:  Vaccine/limited effectiveness  Concerns regarding unknown effects for the child  Unclear cost-benefit ratio | 18  7  5  2 |
| Uncertainties regarding remuneration/restricted choice of influenza vaccine due to contracts between health insurance providers and pharmaceutical companies | 14 |
| Logistical reasons/demand planning | 12 |
| Amount of time required to inform | 11 |
| Contrary views of partners in common practice | 2 |
| Misinformation/Mistaken contraindications (regarding inactivated vaccines):  Immune deficiency  Multiple sclerosis | 2  1  1 |
| Forgetting to talk about vaccination during pregnancy | 1 |
| Communication problems | 1 |

Table S3: Results of logistic regression analysis of variables potentially associated with vaccinating pregnant women against pertussis

|  |  | N (%) | Crude OR  [95%-CI] | p-value | Adjusted^a^ OR  [95%-CI] | p-value |
| --- | --- | --- | --- | --- | --- | --- |
| Performing influenza vaccination in pregnant women | | 846 |  |  |  |  |
|  | No | 74 (8.8%) | Ref |  |  |  |
|  | Yes | 772 (91.3%) | **8.20** [4.43─15.19] | 0.000 |  |  |
| Performing pertussis vaccination in women of child-bearing age | | 847 |  |  |  |  |
|  | No | 106 (12.5%) | Ref |  |  |  |
|  | Yes | 741 (87.5%) | **71.23** [22.37─226.81] | 0.000 |  |  |
| Performing pertussis vaccination in close contacts of infant | | 847 |  |  |  |  |
|  | No | 393 (46.4%) | Ref |  |  |  |
|  | Yes | 454 (53.6%) | **3.53** [2.65─4.71] | 0.000 |  |  |
| Performing pertussis vaccination in women postpartum | | 843 |  |  |  |  |
|  | No | 266 (31.6%) | Ref |  |  |  |
|  | Yes | 577 (68.5%) | **4.80** [3.52─6.55] | 0.000 |  |  |
| Informing pregnant women about on pertussis vaccination | | 848 |  |  |  |  |
|  | No | 151 (17.8%) | Ref |  |  |  |
|  | Only on patient request | 129 (15.2%) | **4.96** [2.17─11.32] | 0.000 |  |  |
|  | Only x years since last vaccination | 302 (35.6%) | **67.81** [31.58─145.62] | 0.000 |  |  |
|  | To all patients | 266 (31.4%) | **110.63** [50.10─244.31] | 0.000 |  |  |
| **(A) reason(s) for not vaccinating pregnant women against pertussis during pregnancy is/are …** | | | | |  |  |
| ...doubts on the effectiveness of the pertussis vaccination | | 831 |  |  |  |  |
|  | Fully or rather agree | 18 (2.2%) | **0.08** [0.18─0.35] | 0.001 | **0.08** [0.18─0.34] | 0.001 |
|  | Partly agree | 22 (2.7%) | 0.64 [0.27─1.45] | 0.298 | 0.69 [0.29─1.65] | 0.403 |
|  | Rather or fully disagree | 791 (95.2%) | Ref |  | Ref |  |
| ...concerns regarding the safety of the pertussis vaccination during pregnancy | | 831 |  |  |  |  |
|  | Fully or rather agree | 97 (11.7%) | **0.10** [0.06─0.17] | 0.000 | **0.11** [0.06─0.19] | 0.000 |
|  | Partly agree | 74 (8.9%) | **0.30** [0.18─0.49] | 0.000 | **0.31** [0.19─0.51] | 0.000 |
|  | Rather or fully disagree | 660 (79.4%) | Ref |  | Ref |  |
| …the low health risk posed by pertussis in infants | | 828 |  |  |  |  |
|  | Fully or rather agree | 21 (2.5%) | 0.60 [0.25─1.44] | 0.253 | 0.61 [0.25─1.49] | 0.275 |
|  | Partly agree | 17 (2.1%) | 0.75 [0.28─1.95] | 0.551 | 0.65 [0.25─1.73] | 0.393 |
|  | Rather or fully disagree | 790 (95.4%) | Ref |  | Ref |  |
| …the lack of a current STIKO vaccination recommendation | | 831 |  |  |  |  |
|  | Fully or rather agree | 334 (40.2%) | **0.12** [0.08─0.16] | 0.000 | **0.11** [0.08─0.16] | 0.000 |
|  | Partly agree | 96 (11.6%) | **0.57** [0.34─0.93] | 0.026 | **0.55** [0.33─0.91] | 0.021 |
|  | Rather or fully disagree | 401 (48.3%) | Ref |  | Ref |  |
| ...difficulties integrating the pertussis vaccination into practice procedures | | 828 |  |  |  |  |
|  | Fully or rather agree | 48 (5.8%) | **0.21** [0.11─0.41] | 0.000 | **0.22** [0.11─0.43] | 0.000 |
|  | Partly agree | 45 (5.4%) | **0.29** [0.15─0.54] | 0.000 | **0.28** [0.15─0.53] | 0.000 |
|  | Rather or fully disagree | 735 (88.8%) | Ref |  | Ref |  |
| …the lack of a monovalent pertussis vaccine | | 830 |  |  |  |  |
|  | Fully or rather agree | 269 (32.4%) | **0.26** [0.19─0.36] | 0.000 | **0.26** [0.19─0.36] | 0.000 |
|  | Partly agree | 141 (17.0%) | **0.56** [0.38─0.84] | 0.005 | **0.57** [0.38─0.85] | 0.006 |
|  | Rather or fully disagree | 420 (50.6%) | Ref |  | Ref |  |
| …the amount of time necessary to inform about the pertussis vaccination | | 831 |  |  |  |  |
|  | Fully or rather agree | 168 (20.2%) | **0.27** [0.19─0.39] | 0.000 | **0.29** [0.20─0.41] | 0.000 |
|  | Partly agree | 143 (17.2%) | **0.62** [0.42─0.90] | 0.013 | **0.61** [0.41─0.89] | 0.011 |
|  | Rather or fully disagree | 520 (62.6%) | Ref |  | Ref |  |
| Sex | | 842 |  |  |  |  |
|  | Female | 640 (76.0%) | Ref |  |  |  |
|  | Male | 202 (24.0%) | **1.51** [1.08─2.10] | 0.015 |  |  |
| Age (in years) | | 828 | 1.00 [0.98─1.01] | 0.664 |  |  |
| Work experience in gynaecological practice (in years) | | 840 | 1.00 [0.99─1.02] | 0.378 |  |  |
| Geographical region | | 835 |  |  |  |  |
|  | West | 692 (82.9%) | Ref |  |  |  |
|  | East | 143 (17.1%) | 1.29 [0.89─1.88] | 0.177 |  |  |
| Influenza vaccination practice of physicians | | 841 |  |  |  |  |
|  | Never | 106 (12.6%) | Ref |  |  |  |
|  | Occasionally | 141 (16.8%) | 1.51 [0.91─2.51] | 0.111 |  |  |
|  | Annually | 594 (70.6%) | **2.15** [1.42─3.26] | 0.000 |  |  |

Abbreviations: CI = confidence interval, OR = odds ratio, STIKO = Standing Committee on Vaccination; n = 807 in adjusted analysis

^a^ adjusted for age, sex, region (east/west)

Table S4: Additional perceived barriers for vaccinating against pertussis during pregnancy provided in participants’ additional comments

| **Categorized barriers** | N |
| --- | --- |
| **Total** | **77** |
| Refusal of pregnant women | 18 |
| Hesitancy of physicians based on:  Concerns regarding unknown effects for the child  Fear of potential legal consequences  Unclear cost-benefit ratio | 15  6  2  1 |
| Amount of time required to inform | 10 |
| Medical reasons:  Contraindications (mostly acute infections and allergies)  Former complications  High-risk pregnancy | 8  5  1  1 |
| Unavailability of vaccine | 7 |
| Insufficient remuneration | 5 |
| Unawareness of possibility of vaccinating pregnant women against pertussis | 5 |
| Contrary to current vaccination strategy:  Vaccination before pregnancy  Vaccination after pregnancy | 5  4  1 |
| Lack of remuneration | 4 |
| Early stage of pregnancy | 2 |
| Vaccination through general practitioner | 2 |
| Forgetting to talk about vaccination during pregnancy | 1 |
| Communication problems | 1 |
| Misinformation/Mistaken contraindications (regarding inactivated Tdap vaccines):  Immune deficiency  Stating the risk of using live vaccine during pregnancy | 2  1  1 |

Table S5: Measures to attain high pertussis vaccination coverage in pregnant women (and high vaccination coverage in general) provided in participants’ additional comments

| **Categorized measures** | N |
| --- | --- |
| **Total** | **117** |
| Inform the public through media:  Use of positive statements  Use of non-scientific media: TV, campaigns, internet, social media/apps  Counter false statements about vaccination and legal consequences (n=4)  Inform in educational settings  Use of role models | 56  48  15  12  6  2 |
| Regulatory changes:  Man vaccination  Improved remuneration for performing vaccinations  Removing ASHIP restrictions to make remuneration for vaccination of contacts possible  Remuneration for consultations itself  Ensure availability of (monovalent) vaccines  Vaccination of children in community facilities  Harmonization of remuneration between federal states | 50  19  14  12  5  4  4  1 |
| Informing and involvement of health care professionals:  Informing and training midwives/involve the associations of midwives  Continuing medical education of physicians, including general practitioners  Increased involvement of general practitioners  Training and information material for supportive staff in medical practice | 19  10  9  6  1 |
| Other specific suggestions:  Vaccination reminder for patients: documentation on health insurance card or vaccination pass  Addressing patients personally and taking sufficient time for consultation  Bonus system by health insurance providers for complete vaccine protection status  Increasing physicians’ motivation and confidence to vaccinate  Mandatory individual vaccination plan in writing  Appropriate health insurance coverage in case of vaccination adverse effects^[[1]](#footnote-2)^ | 13  3  3  2  2  2  1 |

1. This is the case in Germany and is addressed in § 60 of the German Infection Protection Act (IfSG) for vaccinations recommended by the STIKO [↑](#footnote-ref-2)
