# Supplementary material for: Influenza and pertussis vaccination during pregnancy – attitudes, practices and barriers in gynaecological practices in Germany
Source: BMC Health Serv Res. 2019 Sep 2;19:616. doi: 10.1186/s12913-019-4437-y (PMC6719372; doi:10.1186/s12913-019-4437-y)
Supplement: Supplementary file 2 — Translated questionnaire (originally used in German) used for this survey. (PDF 138 kb) [file 12913_2019_4437_MOESM2_ESM.pdf]

This is a translation for scientific purposes of a German-language questionnaire developed by the Robert Koch Institute for a survey among gynaecologists that was conducted in 2017.

GENERAL QUESTIONS REGARDING VACCINATION

1. Do you offer your patients the opportunity to check their vaccination card? (multiple answers possible)
   Yes, at every doctor-patient contact
   Yes, to all new patients
   Yes, during the annual cancer screening examination
   Yes, if there is a wish to have children
   Yes, during pregnancy
   Yes, on request
   No

2. Do you carry out vaccinations against the following pathogens in your practice?

Measles/Mumps/Rubella (MMR)	Yes/No
Varicella	Yes/No
Influenza	Yes/No
HPV	Yes/No
Tetanus/Diphtheria/Pertussis (Tdap)	Yes/No

QUESTIONS ABOUT INFLUENZA VACCINATION

3. Were you aware of the STIKO recommendation for influenza vaccination of pregnant women before this survey?
   Yes/No

4. Do you/your practice team inform your pregnant patients about the influenza vaccination?
   Yes, always / Yes, but only at patient request / No

5. Is there any information material (e.g. notices, flyers) on influenza vaccination for patients in your practice? (multiple answers possible) Yes, generally on influenza vaccination / Yes, especially for pregnant women / No

6. Are you familiar with the information material of the Federal Centre for Health Education (BZgA) on influenza vaccination for pregnant women, such as the campaign "Wir kommen der Flu zuvor"? Yes/No

7. What information material on influenza vaccination for pregnant women does your practice use? (multiple answers possible) None / Those of the BZgA / Others, namely: _______

8. Do you recommend the use of influenza vaccination to pregnant women? Yes/No

   8.1 Do you perform influenza vaccination in pregnant women in your practice? Yes/No (if „No“, continue with 8.2)

   8.1.1 What percentage of pregnant women to whom you offer influenza vaccination is vaccinated in your practice? <10% / 10 – 29% / 30 – 49% / ≥ 50% / Unclear

   8.2 Which influenza vaccine would you prefer for pregnant patients?
   Trivalent vaccine / Tetravalent vaccine / No preference / Uncertain

9. The following statements are possible reasons that could speak <u>against</u> influenza vaccination during pregnancy. Please evaluate the extent to which these apply to you. (Please rate each statement)
   Options: Fully agree / Rather agree / Party agree / Rather disagree / Fully disagree

   A reason for me not to vaccinate pregnant women against influenza...

   ...are doubts about the effectiveness of influenza vaccination.
   ...are concerns about the safety of influenza vaccination during pregnancy.
   ...is the low disease severity of influenza in pregnant women.
   ...are difficulties to logistically incorporate influenza vaccination into practice procedures.
   ...is the time and effort needed to inform about influenza vaccination of pregnant women.
   ...are restrictions through regulations by the Association of Statutory Health Insurance Physicians.
   In your opinion, are there other reasons why pregnant women should not be vaccinated against influenza?

10. Do you recommend influenza vaccination to women who wish to have children? Yes/No

QUESTIONS ABOUT PERTUSSIS VACCINATION

11. Were you aware of the STIKO recommendations for the pertussis vaccination of the following groups of persons before this survey?

    11.1. Women of childbearing age (if the last vaccination was more than 10 years ago) Yes/No
    11.2. Close contacts of infants (if the last vaccination was more than 10 years ago) Yes/No
    11.3. Postpartum (if the last vaccination was more than 10 years ago) Yes/No

12. Do you recommend pertussis vaccination to women of childbearing age if the last pertussis vaccination was more than 10 years ago? Yes/No

12.1. Do you perform pertussis vaccination for women of childbearing age in your practice? Yes/No

13. Do you inform your pregnant patients (if necessary accompanying person, especially partner) about the recommendation to vaccinate close contact persons of the future newborn against pertussis if the last vaccination was more than 10 years ago? Yes/No

13.1. Do you perform pertussis vaccination of partners or other contact persons in your practice? Yes/No
13.2. Do regulations of your Association of Statutory Health Insurance Physicians restrict you in the vaccination of partners or other contact persons? Yes/No

14. If the last pertussis vaccination was more than 10 years ago (i.e. no vaccination given before or during pregnancy), do you recommend pertussis vaccination to your patients during the first days after giving birth? Yes/No

14.1. Do you perform pertussis vaccination postpartum? Yes/No

15. Do you inform your patients about the possibility of pertussis vaccination during pregnancy? (multiple answers possible)
Yes, all
Yes, if more than the following number of years have passed since the last pertussis vaccination: (Please fill in)
Yes, if the patient asks about the vaccination.
No

15.1. Do you perform pertussis vaccination in pregnant women in your practice? Yes/No (if „No“, continue with 16.)

15.1.1. What percentage of pregnant women to whom you offer pertussis vaccination is vaccinated in your practice? <10% / 10 – 29% / 30 – 49% / ≥ 50% / Unclear

16. The following statements are possible reasons that could speak <u>against</u> pertussis vaccination during pregnancy. Please evaluate the extent to which these apply to you. (Please rate each statement)
Options: Fully agree / Rather agree / Party agree / Rather disagree / Fully disagree

A reason for me not to vaccinate pregnant women against pertussis...

...are doubts about the effectiveness of vaccination to prevent pertussis in infants.
...are concerns about the safety of pertussis vaccination during pregnancy.
...is the low disease severity of pertussis in infants.
...is the missing STIKO recommendation.
...are difficulties to logistically incorporate pertussis vaccination into practice procedures.
...is the lack of pertussis-only vaccine (availability only in combination with tetanus and diphtheria).
...is the time and effort needed to inform about pertussis vaccination of pregnant women.
In your opinion, are there other reasons why pregnant women should not be vaccinated against pertussis?

17. Would you generally recommend or offer your pregnant patients the pertussis vaccination if there was a corresponding STIKO recommendation? Yes / No / Don't know

18. Which measures do you consider suitable to achieve high pertussis vaccination coverage, in case STIKO should recommend Tdap vaccination for pregnant women? (Please rate each statement)
Options: Very suitable / Rather suitable / Partly suitable / Rather not suitable / Not suitable at all / Unsure

- Inclusion of vaccination recommendations in the maternity record
- Information material for physicians on vaccination recommendations
- Information material for the practice for vaccination of pregnant women
- Advocate opinion by professional association
- Informing midwives about the usefulness of vaccinations
- Germany-wide information campaign on Tdap vaccination for pregnant women
- Software reminder for vaccinating physicians
- Improved remuneration for informing about and performing vaccinations

In your opinion, are there any other measures that could contribute to a high vaccination coverage?

DEMOGRAPHIC AND GENERAL INFORMATION

19. Sex: Female / Male
20. Age (in years)
21. How long have you been working in (a) gynaecological practice(s)? (in years)
22. Which Association of Statutory Health Insurance Physicians are you affiliated with?
23. Do you obtain influenza vaccination? Yes, annually / Yes, occasionally (not annually) / No
24. Is there anything else you would like to tell us?
